# Specific dietary practices in female athletes and their association with positive screening for disordered eating

**DOI:** 10.1186/s40337-021-00407-7

**Published:** 2021-04-17

**Authors:** Celina de Borja, Bryan Holtzman, Lauren M. McCall, Traci L. Carson, Laura J. Moretti, Nicole Farnsworth, Kathryn E. Ackerman

**Affiliations:** 1 Female Athlete Program, Division of Sports Medicine, Boston Children’s Hospital, 319 Longwood Avenue – 6th Floor, Boston, MA 02115 USA; 2 Department of Orthopaedic Surgery, University of California San Francisco, San Francisco, CA USA; 3 Perelman School of Medicine at the University of Pennsylvania, Philadelphia, PA USA; 4 Department of Epidemiology, University of Michigan School of Public Health, Ann Arbor, MI USA; 5 Neuroendocrine Unit, Massachusetts General Hospital, Harvard Medical School, Boston, MA USA

**Keywords:** RED-S, Eating disorder, Disordered eating, Female athlete

## Abstract

**Background:**

To determine if following specific diets was associated with reporting behaviors that are consistent with disordered eating compared to non-diet-adherent athletes. We hypothesized that athletes adhering to specific diets were more likely to report disordered eating than those not following a diet.

**Methods:**

One thousand female athletes (15–30 years) completed a comprehensive survey about athletic health and wellness. Athletes were asked to specify their diet and completed 3 eating disorder screening tools: the Brief Eating Disorder in Athletes Questionnaire, the Eating Disorder Screen for Primary Care, and self-reported current or past history of eating disorder or disordered eating. Descriptive statistics were calculated for all study measures and chi-squared tests assessed relationships between athletes’ dietary practices and their responses to eating disorder screening tools. Statistical significance was defined as *p* < 0.05.

**Results:**

Two hundred thirty-four of 1000 female athletes reported adherence to specific diets. 69 of the 234 diet-adhering athletes (29.5%) were excluded due to medically-indicated dietary practices or vague dietary descriptions. Of the 165 diet-adherent athletes, 113 (68.5%) screened positively to ≥1 of the 3 eating disorder screening tools. Specifically, athletes practicing a low-carbohydrate diet were more likely to report disordered eating vs. athletes without dietary restrictions (80% vs. 41.8%; *p* < 0.0001).

**Conclusion:**

Specific diet adherence in female athletes may be associated with reporting behaviors that are consistent with disordered eating. Health practitioners should consider further questioning of athletes reporting specific diet adherence in order to enhance nutritional knowledge and help treat and prevent eating disorders or disordered eating.

## Plain English summary

Female athletes may follow special diets for various reasons including sociocultural practices, environmental concerns, or health and weight-management benefits. These practices may put them at risk for developing eating disorders, which peak during adolescence, and are more common among athletes. Eating disorders or disordered eating in athletes may lead to inadequate fueling or low energy availability and its subsequent health and performance-related complications, also known as Relative Energy Deficiency in Sport (RED-S). It may be difficult to detect eating disorders among athletes due to the secretive nature behind these behaviors. In addition, objective information such as low weight or body mass index (BMI) may not be reliable due to their increased muscle mass. The results of this study revealed that female athletes who reported practicing special diets, especially those who follow a low-carbohydrate diet may be associated with developing eating disorders compared to female athletes who did not report any dietary restrictions. These findings should prompt healthcare providers to evaluate these individuals further in order to enhance their nutritional knowledge and help treat and prevent consequences linked to eating disorders or disordered eating.

## Background

Many female athletes choose to follow specific diets [1–3]. Common motivations for these dietary practices may be from religious, social, or environmental concerns [2]. For some athletes, specific diets may be used to manage certain medical conditions, such as celiac disease (gluten free), lactose intolerance (dairy free), or epilepsy (ketogenic). Health, weight-management, weight-class restrictions, and aesthetic requirements for sport may also influence an athlete’s decision regarding specific dietary practices [2, 4, 5]. Claims of performance enhancement by professional or high profile athletes, often propagated on social media, may also encourage young athletes to adopt specific dietary practices despite insufficient scientific evidence [3].

Specific dietary preferences or restrictive diets may predispose athletes to developing eating disorders [4–6]. Eating disorders or disordered eating are more common in athletes than non-athletes [4–7]. A study published in 2004 revealed that the prevalence of eating disorders amongst Norwegian elite athletes was 13.5% compared to 4.6% of the general population [8]. Prevalence of eating disorders is higher in female athletes compared to male athletes, ranging from 0 to 19% for men, and 6–45% for women [8, 9]. This wide range for prevalence rates may be due to large variations in study design and constantly changing diagnostic criteria for eating disorders, that may influence results. Sport-specific risk factors include endurance and aesthetic sports for women, and weight-class sports for men [9]. The incidence usually peaks during adolescence, possibly due to a combination of pubertal changes, social stigmas revealing body image dissatisfaction, and increased intensity and competitiveness of athletic pursuits [10, 11]. Early detection and treatment of athletes with eating disorders or disordered eating can prevent development of serious complications [12]. However, identifying athletes who are at highest risk for developing eating disorders is difficult because of under-reporting of symptoms or eating habits, likely due to the secretive nature and denial surrounding these behaviors [4, 7]. Some authors suggest that engaging in special diets due to various reasons may be used as a socially acceptable way to conceal disordered eating [13, 14]. External factors such as seeking the approval of their coaches or their peers may have also perpetuated these behaviors as normal practices in sport [9]. The most recent updates in the 5th edition of the Diagnostic and Statistical Manual of Mental Disorders (DSM-5) have also made diagnosis of eating disorders somewhat more challenging because some of the objective criteria for diagnosis of anorexia nervosa (e.g., low weight or BMI and amenorrhea) have been modified, and the epidemiology of eating disorders diagnoses may change [15, 16].

Fueling an athlete requires ensuring that basic dietary requirements are met and sports-specific diet-related objectives are achieved. In addition to the nutritional requirements necessary for basic metabolic functions, dietary adjustments should be considered to accommodate variations in training load that occur throughout the different seasons in sports [17, 18]. Ongoing growth and development also need to be factored into nutritional considerations for a young or adolescent athlete [17]. Adolescents experience growth spurts and have higher resting metabolic rates compared to adults, highlighting the importance of increased caloric intake during this period [19, 20]. Along with caloric consumption, it is also extremely important for adolescent athletes to consume adequate amounts of calcium and vitamin D for optimal skeletal health [21]. Physical changes experienced by female athletes during adolescence include increased percent body fat and decreased percent lean muscle mass, which may lead to body image dissatisfaction, especially among those who participate in aesthetic or weight-sensitive sports. The combination of these physical and psychosocial factors that occur around puberty may be a deterrent for adolescent female athletes to maintain adequate nutrition during this crucial period of growth and development [9].

A concern with restrictive diets and eating disorders or disordered eating in athletes is that inadequate education or poor understanding of an athlete’s nutritional and energy requirements may lead to low energy availability, and consequently some of the various negative sequelae of Female Athlete Triad and Relative Energy Deficiency in Sport (RED-S). These may include but are not limited to menstrual dysfunction, impaired bone health and metabolism, increased injury risk, decreased endurance, performance and coordination [22–24].

Detection of eating disorders or disordered eating in adolescents is difficult, yet prognosis is better the earlier they are detected and treated. Thus, determining other risk factors or markers of eating disorders are important. In this study, we hypothesized that athletes who report adhering to special diets were more likely to report disordered eating when compared to non-diet adherent athletes.

## Methods

### Participants

Female athletes, 15–30 years of age, completing ≥4 hours of self-reported physical activity per week for at least 6 months, and seen at the sports medicine clinic at Boston Children’s Hospital, were invited to participate in the survey. The eligible age group was chosen to represent a population of competitive high school, collegiate, and postgraduate athletes of reproductive age. Non-athletes, males, and female athletes not able to participate in sports (e.g., due to injury) within the past 6 months were excluded from the study. The Institutional Review Board (IRB) at Boston Children’s Hospital approved this study. Informed consent was obtained from participants who were ≥ 18 years of age and parents of those < 18 years of age; assent was obtained from participants < 18 years of age.

### Anthropometric measurements

Heights and weights of participants were collected as part of their clinic visits. Heights were measured to the nearest 0.1 cm with a wall-mounted or free-standing stadiometer depending on clinic site. Weights were measured on medical electronic scales to the nearest 0.1 kg. Participants were weighed in light clothing with no footwear.

### Survey

A detailed description of the survey has been reported previously [22]. The survey was administered through an online questionnaire, which included 133 questions pertaining to general health, illness, injury, sports performance and Triad/RED-S risk factors.

#### Assessment of dietary practices

Patients were asked if they followed a specific diet or if they were avoiding certain types of foods/food groups, and to specify which dietary practice they were following. These questions are displayed in Table 1. Athletes following diets in association with specific health issues mentioned in their medical history (e.g., gluten free due to diagnosed celiac disease, dairy free for lactose intolerance) or vague dietary descriptions that did not fall into any of the main categories (e.g., “eating healthy,” “exclude junk”) were categorized separately and excluded from the analysis.

**Table 1:** Summary of eating disorder screening tools used in the survey

Dietary practices	BEDA-Q ≥ 1 [25]	ESP [26]	Self-report
• Are you on a special diet or do you avoid certain types of foods or food groups? ^b^ • Please explain what type of diet you are on (e.g. Gluten free/ Dairy free/ Vegetarian/ Low carb)	• I feel extremely guilty after overeating^a^ • I am preoccupied with the desire to be thinner^a^ • I think that my stomach is too big^a^ • I feel satisfied with the shape of my body^a^ • My parents have expected excellence of me^a^ • As a child, I tried very hard to avoid disappointing my parents and teachers^a^ • Are you trying to lose weight now?^b^ • Have you tried to lose weight? ^b^ • If yes, how many times have you tried to lose weight?^c^	• Are you satisfied with your eating patterns?^b^ • Do you ever eat in secret?^b^ • Does your weight affect the way you feel about yourself?^b^ • Do you currently suffer with or have you ever suffered in the past with an eating disorder?^b^	• Do you or have you ever suffered from disordered eating?^b^ • Do you currently suffer with or have you ever suffered in the past from an eating disorder?^b^

Brief Eating Disorder in Athletes Questionnaire (BEDA-Q)

Eating Disorder Screen for Primary Care (ESP)

^a^Answer choices: always, usually, often, sometimes, rarely never

^b^Answer choices: yes, no

^c^Answer choices: 1–2, 3–5, > 5 times

#### Eating disorder assessment

Two validated eating disorder questionnaires, the Brief Eating Disorder in Athletes Questionnaire (BEDA-Q) and the Eating Disorder Screen for Primary Care (ESP), and self-reported current or past history of eating disorder or disordered eating were included in the survey. The BEDA-Q is a validated screening tool for eating disorders specific for female athletes with a sensitivity of 82.1% and specificity of 84.6% for detecting ED. [25] The ESP is a four-item questionnaire used in the primary care setting wherein responding yes to ≥3 questions is associated with an increased risk of eating disorders [likelihood ratio (95% CI) = 11 (6.4–18))] [26]. The questions for each tool are displayed in Table 1.

### Study design

This study is a secondary analysis of preexisting data from a large-scale cross-sectional survey [22]. This study explores the correlation between the specific dietary practices of the participating female athletes and eating disorders. Data set is owned by the researchers and a separate codebook was created to gather variables that are relevant for this study.

### Data analysis

Statistical analyses were performed using the R Studio statistical package (version 1.1.453). Descriptive statistics were calculated for all study measures. We ran chi-squared tests with a Bonferroni correction to account for multiple comparisons between athletes’ dietary practices and their responses to eating disorder screening tools. Statistical significance was set at *p* < 0.05.

## Results

### Sample characteristics

1000 female athletes completed the survey and were included in the parent study (age mean ± SD, 18.92 ± 3.34 years) relating eating disorder screening and RED-S correlates [22]. As previously reported, out of 1000 female athletes who participated in this study, 473 (47.3%) screened positively to at least one of the three eating disorder screening tools [22]. 234 (23.4%) of participants reported adhering to specific diets; 69 of these athletes (29.5% of subset; 6.9% of total sample) reported that their specific diet was medically indicated (*n* = 27) or had vague dietary descriptions that did not fall into any of the main categories (*n* = 42).

The mean age (19.80 years ±3.50) of athletes who reported adherence to non-medically indicated specific dietary practices was slightly higher compared to those who were not on any special diets. Mean weight (63.05 kg ±13.60) was also slightly higher for the diet adherent group, but mean BMI and height were similar for both groups. Descriptive characteristics of each group are displayed in Table 2.

**Table 2:** Descriptive characteristics of each group. Mean ± SD

	No dietary preference (***n*** = 766)	Reported dietary preference (***n*** = 165)	Excluded due to medically-indicated or non-specific dietary preference (***n*** = 69)
**Age (years)**	18.66 ± 3.20	19.80 ± 3.50	21.45 ± 3.40
**BMI (kg/m**^**2**^**)**	22.90 ± 3.70	22.86 ± 3.74	22.70 ± 3.46
**Height (cm)**	165.20 ± 6.92	165.13 ± 6.41	166.07 ± 7.05
**Weight (kg)**	62.54 ± 11.16	63.05 ± 13.60	63.01 ± 12.97

### Dietary practices among female athletes

**Fig. 1:**
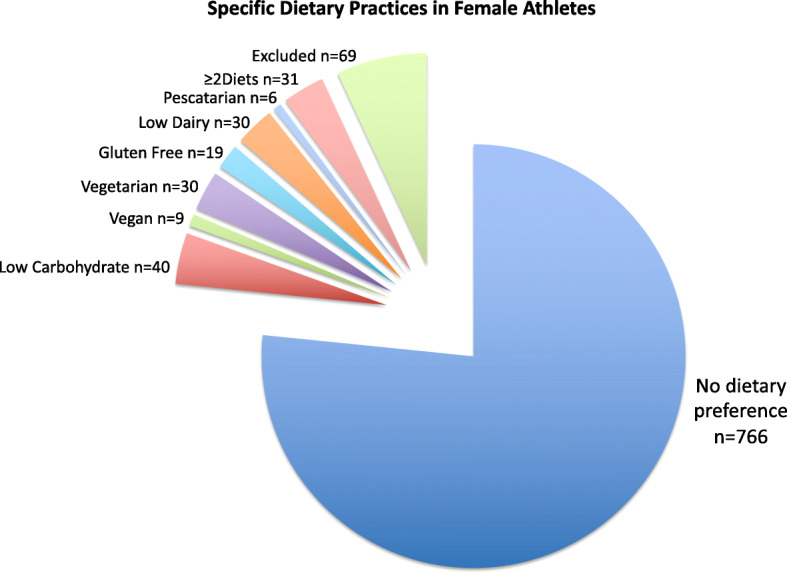
Specific dietary preferences in female athletes

Of the 165 athletes who were diet-adherent without medical indication, 81.2% reported following only one diet and 18.8% reported following ≥2 diets. Of the 134 athletes who reported following only one diet, the most common dietary practices were: low carbohydrate (29.8%), vegetarian (22.5%), dairy restrictive (22.5%), gluten free (14%), vegan (6.7%), and pescatarian (4.5%). The distributions of specific dietary practices in female athletes are displayed in Fig. 1.

### Dietary practices and response to eating disorder screening tools

**Fig. 2:**
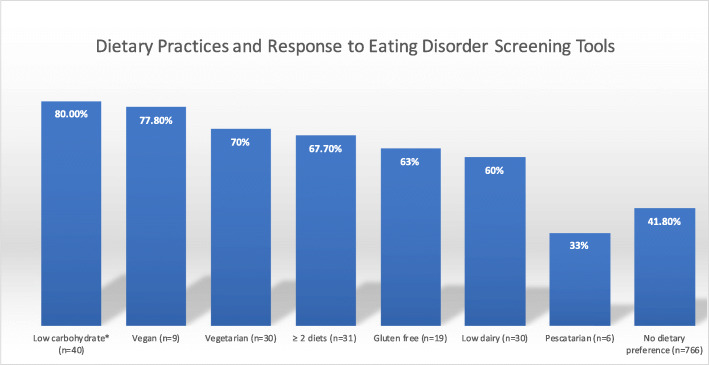
Dietary practices and response to eating disorder screening tools. *p-value< 0.0001

Of all the diet-adherent athletes included in the analysis (*n* = 165), 68.5% screened positively to ≥1 of the 3 eating disorder screening tools compared to 41.8% of the non-diet-adherent athletes (*n* = 766) (*p* < 0.0001) Athletes practicing a low-carbohydrate diet were more likely to report disordered eating when compared to athletes without dietary restrictions (80% versus 41.8%) (*p* < 0.0001, Table 3). Athletes following a vegan, vegetarian, gluten-free, low-dairy, or ≥ 2 diets resulted higher rates of reporting disordered eating, however, did not yield statistical significance when compared to athletes without dietary restrictions. Athletes following a pescatarian diet resulted lower rates of reporting disordered eating compared to those who were not diet-adherent. Percentage of athletes who screened positively on eating disorder screening tools are displayed in Fig. 2.

**Table 3:** Specific dietary practices and correlation with eating disorder screening tools. * as compared to athletes without dietary restrictions

Dietary Practice	Screened positively on eating disorder screening tools	*p-value
Low carbohydrate *n* = 40	80.0%	< 0.0001
Vegan *n* = 9	77.8%	0.4081
Vegetarian *n* = 30	70.0%	0.0602
≥ 2 diets *n* = 31	67.7%	0.1092
Gluten free *n* = 19	63.2%	0.9807
Low dairy n = 30	60.0%	0.9128
Pescatarian n = 6	33.3%	1

### Eating disorder screening tools

**Fig. 3:**
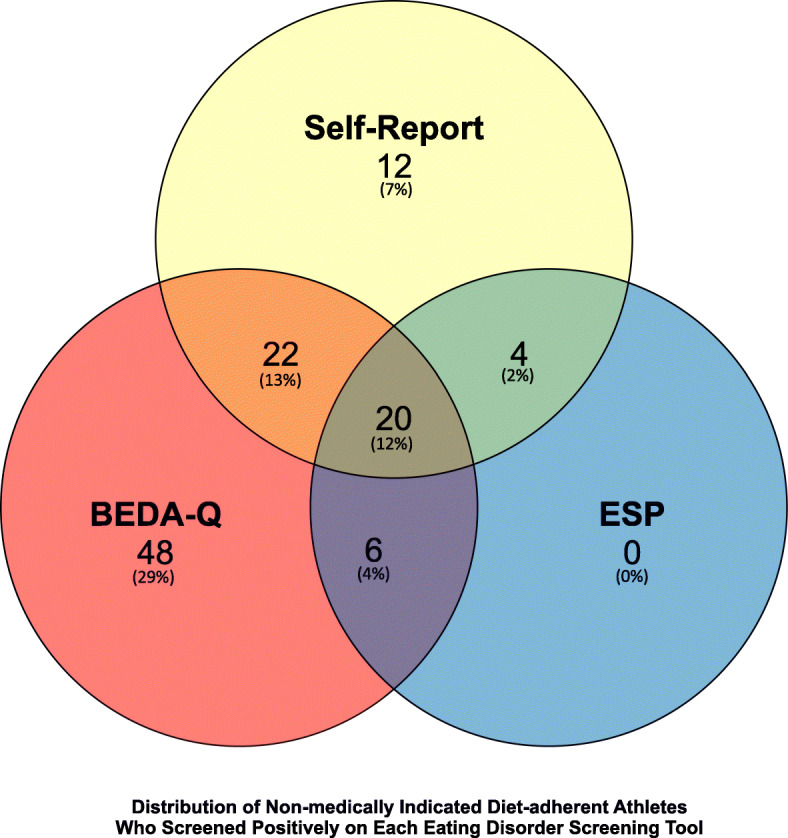
Distribution of non-medically indicated diet-adherent athletes who screened positively on each Eating Disorder screening tool. Brief Eating Disorder in Athletes Questionnaire (BEDA-Q) Eating Disorder Screen for Primary Care (ESP)

29.1% of diet-adherent athletes screened positively on the BEDA-Q alone, while 7.3% screened positively by self-report. None of the diet-adherent athletes screened positively on ESP alone. Overlapping areas represent the subset of athletes who screened positively on 2 or more screening tools. 12.1% of athletes screened positively on all three screening tools (Fig. 3).

## Discussion

Special diets or food restrictions are common in athletic populations [1–3]. Restrictive dietary adherence amongst athletes have been recognized as a risk factor for low energy availability and its subsequent RED-S health and performance-related consequences [4–6]. However, we are unaware of any studies that have examined the relationship between specific diets and eating disorders in young athletic populations. Our study shows that non-medically indicated diet-adherent athletes are more likely to report behaviors that are consistent with disordered eating compared to non-diet-adherent athletes.

Low-carbohydrate diets have been around for decades; however, this dietary practice remains poorly defined. Individuals adhering to a low-carbohydrate diet may vary from simply avoiding these food groups, reducing the amount of daily carbohydrate intake (< 100 g/day), to severely restricting the amount of daily carbohydrate intake (< 50 g/day) to induce ketosis [27, 28]. Recommended macronutrient intake for female athletes range from 3 to 10 g/kg of the athlete’s body weight per day for carbohydrates, 1.2–2.0 g/kg/day for protein, and 20–35% of total kcal/day for fat [2]. Our study found that out of the various dietary practices that athletes may adhere to, those that follow a low-carbohydrate diet without any clinical indications were more likely to report disordered eating than non diet-adherent athletes. It would be difficult to conclude causal mechanisms between these specific diets and development of eating disorders without knowledge of the athlete’s motivation behind these behaviors or compliance with their respective dietary practices. The cross-sectional design of our study also makes it impossible to determine the sequence between dietary adherence and behaviors associated with eating disorders. It is possible that restricting certain macronutrients, particularly carbohydrates, may be a common method that athletes utilize in their desire to manage or lose weight, which may suggest why adhering to such dietary practice may be used as an early indicator of developing eating disorders. Without proper guidance on how to account for recommended dietary intake, these diet-adherent athletes may fail to consume sufficient levels of these macronutrients and may be at risk for RED-S; high-risk consequences specifically include impaired glycogen and muscle protein synthesis and bone remodeling, which may affect how athletes recover from exercise and their overall bone health [2].

A gluten-free diet restricts food that contain the protein gluten, commonly in wheat, rye, or barley. Gluten-free diets are clinically indicated in managing gastrointestinal symptoms in patients who have celiac disease or non-celiac gluten sensitivity [2, 3]. A dairy-free diet restricts the intake of milk-derived products and are medically indicated for lactose intolerant individuals. In addition to medical indications or health and weight management benefits, popular trends initiated by professional or high profile athletes may have also driven young athletes to participate in specific diets despite the absence of scientific evidence of performance benefits [3–5]. Recent studies have shown that the majority of athletes practicing gluten-free diets have chosen to do so based on self-diagnosis, and despite lacking substantial evidence to support performance benefits, there has been an increase in the prevalence of non-celiac, non-gluten sensitive athletes adopting a gluten-free diet in attempt to optimize health and enhance athletic performance [3]. This behavior is somewhat apparent in our study, as some female athletes have reported restricting gluten and dairy from their diets without reporting any medical conditions. Gluten-free diets may be associated with suboptimal intake of carbohydrates and protein and can lead to micronutrient deficiencies (e.g., B vitamins, calcium, vitamin D, iron, and potassium), while low-dairy diets may be deficient in calcium, vitamin D and fat [2, 3].

Plant-based diets may be differentiated based on their restriction of certain food groups [2]. A pescatarian diet excludes meat and poultry but includes fish, dairy and eggs; a lacto-ovo vegetarian diet excludes meat, poultry, and fish, but includes eggs and dairy; a vegan diet excludes all meat, poultry, fish, eggs, and dairy [2]. Our findings suggest that vegetarian athletes who avoided more food groups resulted higher rates of reporting disordered eating, in contrast to athletes who only avoided meat/poultry and practiced a pescatarian diet. However, results were only trending on significance, hence, more research is necessary to better understand the relationship between restriction of specific food groups within the different plant-based diets and development of eating disorders. Plant-based diets have been linked to lower body mass index (BMI) and decreased risk of chronic disease, such as hypertension, diabetes, and obesity [2, 29]. Although plant-based diets have been found to offer significant health benefits, these diets with high fiber content can cause early satiety and appetite blunting. These effects, in turn, if without proper guidance on adequate macronutrient or micronutrient intake may lead to low energy intake amongst young and highly athletic individuals, and subsequently low energy availability and its associated RED-S health and performance consequences [2, 30]. In addition, individuals practicing plant-based diets who do not consume dairy products or fish may be at risk for vitamin D and calcium deficiency, which are vital for maintaining adequate bone health [2, 29–31].

Previous studies have found a positive correlation between special diets and disordered eating [13, 14, 32–35]. Specifically, patients diagnosed with anorexia nervosa have a higher prevalence of practicing vegetarianism at some point in their lives compared to the general population [32, 33]. Although there may be many reasons for special dietary practices, researchers have suggested that adherence to dietary restrictions may be used as a socially acceptable way to disguise disordered eating habits or behaviors [13, 14].

Eating disorders are more prevalent among athletes compared to their non-athletic peers [4–7]. Peak onset is usually around puberty or adolescence (15–19 years old) [11] possibly due to the combination of biological and psychosocial changes that occur, which may result in issues with body dissatisfaction, self-esteem, and mood [10]. Objective information, such as low weight and BMI, may not be reliable among athletes due to their increased muscle mass, wherein energy deficient athletes can present with normal BMIs [4, 7]. Indeed, in our parent study, the group with inadequate energy availability had a higher BMI than the group with adequate energy availability [22]. In the present study, however, we found an association between athletes reporting specific dietary practices and reporting behaviors that are consistent with disordered eating. Additionally, we found that individual athletes screened positively on some eating disorder screening tools and negatively on others, highlighting that it is may be clinically important to ask athletes about eating behaviors with various questions, framing the questions slightly differently to detect eating disorders or disordered eating.

Early detection of eating disorders or disordered eating among athletes is highly recommended to facilitate prompt treatment and prevention of health and performance-related consequences [4, 36] such as menstrual dysfunction, impaired bone health and metabolism, cardiac arrhythmias/abnormalities, endocrine/metabolic dysfunction, increased injury risk, decreased endurance, performance, and coordination [12, 22–24, 37]. The findings in this study may help bridge the gap in recognizing athletes at risk for developing eating disorders. The presence of specific dietary practices in female athletes should urge healthcare providers to consider further evaluation through validated eating disorder questionnaires even before the presence of subjective (e.g., desire to lose weight, body image dissatisfaction) or objective findings (e.g., low weight/BMI, oligomenorrhea) that are commonly used to diagnose eating disorders. Applying this in clinical practice may encourage healthcare providers to address behaviors linked to eating disorders or disordered eating in athletes, discuss their health implications, and offer access to appropriate resources, including nutritional and psychological support. Management of eating disorders or disordered eating in the athletic population requires a thorough evaluation and an interdisciplinary approach that focuses on patient-centered care [2, 7, 38]. A registered dietitian who is experienced in treating athletes with eating disorders may help with managing nutritional adjustments, safe and effective supplementation, and monitoring of energy intake [2, 7, 36, 38]. In our study, the gluten-free diet for celiac disease and dairy-free diet for lactose intolerance were the most common medically-indicated special diets. In these cases, it is important for healthcare providers to educate these athletes regarding the possible macronutrient and micronutrient deficiencies associated with these diets that can lead to low energy availability, and guide them towards proper adjustments with nutritional intake or supplementation [2]..

There were several limitations to this study. This study was a survey, with inherent biases associated with self-report compared to direct food monitoring. This study was a secondary analysis, which only included a small sample size of each of the dietary practices included in the analysis. The cross-sectional study design limits our ability to conclude order of events between special diets and eating disorders. Questions pertaining to the athlete’s motivation behind these dietary adherences (e.g., socio-cultural, weight management, performance benefits, etc.), as well as the duration and compliance surrounding these practices were not included in the survey. To our knowledge, this study is the first to explore the association between various dietary practices and eating disorders specifically in adolescent and young adult female athletes, who are considered high risk for these conditions. Our findings reflect a phenomenon that is commonly seen in clinical practice that is often considered anecdotal. This study opens the opportunity for a larger study of athletes with each of the specific dietary practices highlighted in this paper, which would improve statistical power and may elucidate possible associations between such restrictive diets and the various health and performance-related consequences of RED-S. Additional data that can objectively characterize extent of restrictive dietary practices may also help clarify the causal mechanism behind specific dietary preferences and risk for development of eating disorders.

## Conclusion

Eating disorders or disordered eating are not uncommon among the athletic population and the behaviors peak during adolescence. Identification of athletes who will eventually develop eating disorders is difficult due to under-reporting of eating habits and often limited objective physical and laboratory measurements to detect eating disorders early on. Gaps in knowledge among healthcare providers and athletic staff may also leave these issues undiagnosed or inappropriately managed [39]. This study suggests that specific restrictive dietary practices in female athletes may be associated with eating disorder symptomology. This is worrisome, because athletes with eating disorders or disordered eating may be at risk for low energy availability and consequently health and performance-related consequences of RED-S. Thus, healthcare providers should consider further questioning of athletes reporting specific dietary practices in order to enhance their nutritional knowledge and help treat and prevent consequences linked to eating disorders or disordered eating.

## Data Availability

The datasets used and/or analyzed during the current study are available from the corresponding author on reasonable request.

## Abbreviations

BMI: Body Mass Index
RED-S: Relative Energy Deficiency in Sport
BEDA-Q: Brief Eating Disorder in Athletes Questionnaire
ESP: Eating Disorder Screen for Primary Care
DSM 5: 5th edition of the Diagnostic and Statistical Manual of Mental Disorders
